# Cartilage to bone transitions in health and disease

**DOI:** 10.1530/JOE-13-0276

**Published:** 2013-10

**Authors:** K A Staines, A S Pollard, I M McGonnell, C Farquharson, A A Pitsillides

**Affiliations:** 1 Comparative Biomedical Sciences, The Royal Veterinary CollegeRoyal College Street, London, NW1 0TUUK; 2 Roslin Institute and R(D)SVS, The University of EdinburghEaster Bush, Midlothian, Edinburgh, EH25 9RGUK

**Keywords:** bone, cartilage, osteoarthritis, chondrocyte, endochondral ossification

## Abstract

Aberrant redeployment of the ‘transient’ events responsible for bone development and postnatal longitudinal growth has been reported in some diseases in what is otherwise inherently ‘stable’ cartilage. Lessons may be learnt from the molecular mechanisms underpinning transient chondrocyte differentiation and function, and their application may better identify disease aetiology. Here, we review the current evidence supporting this possibility. We firstly outline endochondral ossification and the cellular and physiological mechanisms by which it is controlled in the postnatal growth plate. We then compare the biology of these transient cartilaginous structures to the inherently stable articular cartilage. Finally, we highlight specific scenarios in which the redeployment of these embryonic processes may contribute to disease development, with the foresight that deciphering those mechanisms regulating pathological changes and loss of cartilage stability will aid future research into effective disease-modifying therapies.

## Introduction

The transition of cartilage to bone is the basis by which all long bones form. This transition is tightly regulated to ensure both permissive foetal development through endochondral ossification and postnatal longitudinal growth at the epiphyseal growth plate. Tighter management of these dynamic events may contribute to the inherent stability of some cartilage, such as articular cartilage, and this possibility is supported by emerging evidence reporting the aberrant redeployment of these transient events in the inherently stable cartilage in several pathological states such as osteoarthritis. It is therefore plausible that lessons can be realised from the molecular mechanisms underpinning the physiological transient chondrocyte differentiation and function. This knowledge may help to decipher those mechanisms surrounding joint pathology and ossification, and aid future research into effective disease-modifying therapies.

In this review, we discuss aspects of cartilage and bone physiology, as well as the complex cellular processes involved in the cartilage to bone transition during endochondral ossification and at the postnatal growth plate. Moreover, we will compare this transient cartilage phenotype with the stable phenotype adopted by articular chondrocytes in order to ostensibly safeguard their permanence, and will discuss disease pathology in which the aberrant redeployment of the transient phenotype is observed.

## Transient cartilage

During mammalian embryological skeletal development, the appendicular skeleton comprising the pectoral girdle, pelvis and the limbs, arises from the lateral plate mesoderm (Saito *et al*. 2006). These structures are formed by means of endochondral ossification as does much of the axial skeleton including the vertebrae and ribs, which arise from somites of paraxial mesoderm origin.

Ossification processes are regulated during embryological development to produce the wide range of skeletal forms that we see in different animal species. A major challenge in skeletal morphogenesis is the precision of growth regulation that is required to create functional skeletal proportions, which can vary so widely between species, and to produce paired skeletal elements which are near identical in size. Therefore, there are major questions in embryological skeletal development, including how do condensations get to be the correct size and how is directional growth achieved in limb patterning?

The apical ectodermal ridge and the zone of polarising activity control proximo–distal and anterior–posterior patterning in the growing limb bud. These two centres are regulated by signalling pathways such as Indian hedgehog (IHH) and WNT/β-catenin (reviewed elsewhere; Summerbell *et al*. (1973), Kronenberg (2003), Tickle (2003), Towers & Tickle (2009) and Wolpert (2010)). Recent emerging evidence has suggested that planar cell polarity, a non-canonical WNT pathway involving the cadherins Fat and Dachsous (Fat/Dchs), is also important in embryonic skeletal development (Goodrich & Strutt 2011). Gradients of Dchs expression appear to regulate cell shape and directional movement during limb morphogenesis and growth (Dabdoub *et al*. 2003, Vladar *et al*. 2009, Wada & Okamoto 2009, Romereim & Dudley 2011). There is also substantial evidence, from pharmacological immobilisation of embryonic chicks, that movement-induced mechanical bone loading regulates longitudinal growth of skeletal elements (Osborne *et al*. 2002, Lamb *et al*. 2003, Pitsillides 2006). Thus, mechanical stimuli may exert epigenetic control over skeletal development to act as an additional source of phenotypic variation during development.

### Endochondral ossification

**Figure 1:**
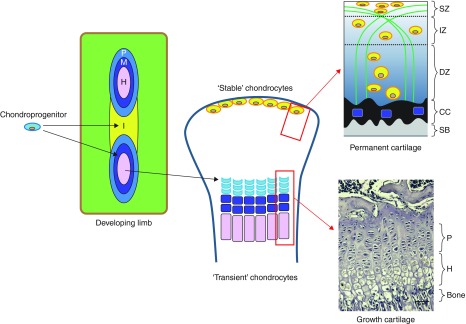
Schematic representation depicting the proposed developmental origins of articular and growth plate cartilage. Mesenchymal aggregation of chondro-progenitors forms the cartilage anlagen and stages of chondrocyte proliferation (P, light blue), maturation (M, purple) and hypertrophy (H, pink) emerge to provide the origins of the future ‘transient’ chondrocytes of the growth plate cartilage. Intervening regions of progressively condensing mesenchyme define the interzone (I, yellow) regions; the position of the future joint and origins of the ‘stable’ articular cartilage chondrocytes. Images (right hand side) depicting the organisation of the mature growth plate (lower) and articular cartilage (upper), consisting of uncalcified cartilage zones (superficial (SZ), intermediate (IZ) and deep (DZ)), and the related chondrocyte and collagen fibril arrangement. The tidemark separates the non-calcified cartilage from the calcified cartilage (CC) which overlies the subchondral bone (SB). Bar=0.1 mm.

The majority of the skeleton is first formed during embryonic development as cartilage anlagen; models of future skeletal elements. The transient nature of this cartilage is reflected in its eventual replacement by bone in the fundamental process of endochondral ossification. Endochondral ossification is initiated by embryonic mesenchymal cells migrating to form pre-cartilage condensations, which then undergo differentiation into chondrocytes and secrete an extracellular matrix rich in collagen type II and aggrecan. The chondrocytes of these cartilage condensations undergo an ordered and highly regulated process involving predominant marginal proliferation and central maturation, hypertrophy and cell death. These latter events define the framework of the endochondral bone and are regulated by paracrine signals including parathyroid hormone (PTH)-related peptide and IHH from the perichondrium (Fig. 1; Storm & Kingsley 1996, Tickle & Münsterberg 2001, Wang *et al*. 2001, Kronenberg 2003, Davey *et al*. 2006, Villavicencio-Lorini *et al*. 2010, Mackie *et al*. 2011).

Primary ossification originates in the centre of the diaphysis of the developing skeletal element. The growth cartilage is invaded by blood vessels, seemingly attracted by VEGF expression by hypertrophic chondrocyte (Zelzer *et al*. 2002) and with this, infiltration of bone-resorbing osteoclasts and bone-forming osteoblasts occurs. Furthermore, the perichondrium becomes vascularised around the bone-forming element to create the periosteum. This process of blood vessel invasion facilitated by degradation of the calcified cartilage extracellular matrix around the hypertrophic chondrocytes is critically reliant on the activity of matrix metalloproteinase 13 (MMP13; Stickens *et al*. 2004). Cartilage matrix resorption is followed by the invasion of osteoblasts, which lay down the newly formed bone. This process spreads longitudinally from the primary ossification centre towards the ends of the bone. Eventually, a secondary ossification centre forms, retaining a cartilaginous growth plate between each epiphysis and the primary ossification centre (Mackie *et al*. 2008).

### Morphology of the postnatal growth plate

The growth plate is a highly specialised developmental region, responsible for postnatal linear bone growth. It consists of chondrocytes, arranged in columns that parallel the axis of the bone, surrounded by their matrix; the precise composition of which is dependent upon the state of chondrocyte differentiation (Fig. 1; Ballock & O'Keefe 2003, Mackie *et al*. 2011). These chondrocytes undergo a series of tightly regulated differentiation and maturation processes, as is reflected by their changing morphology and matrix production, whilst maintaining their spatially fixed locations (Hunziker *et al*. 1987).

Interference with the maturational progression of growth plate chondrocytes leads to abnormal cartilage formation and ossification and modifications in the rates of bone formation, for example, in rickets and dwarfism. Details of the many syndromes and diseases and possible therapeutic strategies are out with the scope of this review but have been the subject of discussion in other reviews (Kornak & Mundlos 2003, Krakow & Rimoin 2010).

The primary zone of the growth plate, often known as the ‘resting’ or ‘germinal’ zone, consists of undifferentiated chondrocyte progenitors. Unlike the remainder of the growth plate, the chondrocytes of the resting zone are distributed sporadically and have a low rate of proliferation (Hunziker *et al*. 1987, Ballock & O'Keefe 2003, Mackie *et al*. 2011). As chondrocytes progress from the resting zone, they gain a proliferative phenotype and adopt a flattened, oblate shape, arranging themselves into longitudinal columns. It is proposed that the creation of these highly organised columns is directed by the chondrocytes in the resting zone, which have been postulated to produce a growth plate-orientating factor (Hunziker *et al*. 1987, Abad *et al*. 2002). The high mitotic activity of these proliferating chondrocytes is controlled by numerous factors, including endocrine/autocrine regulation, circadian rhythm and age. These chondrocytes also undergo a period of high secretory activity as they produce a collagen type-II- and proteoglycan-rich matrix, which with some otherwise marked distinctions closely resembles that seen in the articular cartilage (Farnum *et al*. 2002).

The chondrocytes undergo major phenotypic changes as they gradually become hypertrophic however, until recently the mechanisms by which this occurs is unclear but may involve membrane transporters in the chondrocyte membrane (Bush *et al*. 2008, 2010, Loqman *et al*. 2013). Furthermore, a paper by Cooper *et al*. has defined three distinct phases through which mammalian chondrocyte volume increases: i) true hypertrophy, indicating a proportionate increase in dry mass production and fluid uptake, responsible for approximately threefold increases in volume, ii) a fourfold enlargement solely by cell swelling and iii) a second distinct phase of true hypertrophy with proportionate dry mass and fluid volume increases. These authors show that this final stage is dependent upon insulin-like growth factor 1 (IGF1), a well-recognised regulator of longitudinal bone growth and chondrocyte hypertrophy (Wang *et al*. 1999, Ahmed & Farquharson 2010, Cooper *et al*. 2013, Farquharson & Ahmed 2013). More recent emerging evidence has detailed the regulation of IGF1 signalling in chondrocyte hypertrophy by the WNT signalling pathway, more specifically by WNT-induced secreted protein 3 (Rao *et al*. 2013). Indeed, it appears that the WNT pathway is critical in both growth plate and articular cartilage function, as we have previously discussed in our recent review (Staines *et al*. 2012*a*).

As well as changes in cell morphology, collagen type-II expression decreases during chondrocyte maturation and the hypertrophic chondrocyte initiates the synthesis of collagen type-X (Schmid & Linsenmayer 1985, Kronenberg 2003). The matrix also includes chondrocalcin, osteonectin and osteopontin, as well as having increased membrane activity of alkaline phosphatase, indicating the final maturation phase (Sommer *et al*. 1996, Shen 2005, Roberts *et al*. 2007). Together these changes facilitate mineralisation of the matrix, a complex process reliant upon the levels of calcium and phosphate (Castagnola *et al*. 1988). Mineralisation of the extracellular matrix also enables vascular invasion, through which osteoclasts and osteoblasts gain access via migration to replace the cartilage template with bone (Gerber *et al*. 1999, Horner *et al*. 1999, Engsig *et al*. 2000, Zelzer *et al*. 2002). Recent evidence has suggested that the terminally differentiated hypertrophic chondrocyte may sense signals that initiate this transformation of the cartilage matrix into bony trabeculae through the expression of receptor activator of nuclear factor-κB ligand, an essential cytokine for osteoclast development, function and survival (Xiong *et al*. 2011).

The fate of the terminally differentiated chondrocyte at the chondro-osseous junction is still a matter of debate. However, it is well accepted that this chondrocyte does require to be removed so as to maintain the steady-state thickness of the growth plate. There has been significant evidence to suggest that the chondrocytes die by apoptosis and this appears the most accepted mechanism (Magne *et al*. 2003, Shapiro *et al*. 2005). Indeed the activation of caspases and the decreased expression of the anti-apoptotic factor, Bcl2, in the hypertrophic chondrocytes certainly support this (Amling *et al*. 1997, Adams & Shapiro 2002). The distinct lack of typical apoptotic morphological changes in the terminal hypertrophic chondrocytes does, however, challenge this theory (Emons *et al*. 2009, Carames *et al*. 2010). It would be expected that the condensation of cellular chromatin and the fragmentation of the cell nucleus would be visible, associated with the eventual break down of the cell into several vesicles which are then phagocytosed. Instead, the presence of autophagic vacuoles and the expression of autophagy-regulating genes by growth plate chondrocytes suggest that these cells undergo processes more similar to autophagy than apoptosis (Roach & Clarke 2000, Shapiro *et al*. 2005). Furthermore, the transdifferentiation of chondrocytes has also been proposed (Descalzi Cancedda *et al*. 1995, Roach 1997). This involves the division of the terminal hypertrophic chondrocyte to produce one daughter cell which undergoes apoptosis and one which transdifferentiates into an osteoblast phenotype. However this theory has yet to be fully ratified and would certainly benefit from experiments which use GFP-Cre systems to track the collagen type-X expression of the hypertrophic chondrocyte during its terminal fate.

Longitudinal bone growth occurs at the growth plate until it closes once sexual maturity has been reached. This is initiated through the formation of mineralised tethers between epiphyseal and diaphyseal bone promoting the fusion of the primary and secondary ossification centres (Haines 1975). The chondrocytes of the growth plate reach a state of senescence as they exhaust their proliferative potential, and longitudinal bone growth is ceased. In humans, oestrogen mediates these effects in both males and females and the processes controlling fusion are relevant to understanding the ‘permanent’ loss of this transient chondrocyte phenotype (Nilsson *et al*. 2005, Mackie *et al*. 2011).

## Stable cartilage

### Articular cartilage formation

The earliest emergence of ‘stable’, articular cartilage during skeletal patterning occurs with the formation of interzones; regions of undifferentiated mesenchyme which separate developing skeletal elements in which the joint will later form. There has been some debate as to whether individual skeletal elements are discrete from the outset of their development, or whether these elements form from a single cartilage condensation which is later divided by interzones (Fig. 1; Pitsillides & Ashhurst 2008).

Transient growth cartilage and articular cartilage differ in their collagen composition during their development; in embryonic growth cartilage, expression of collagen type-IIA precedes collagen type-IIB expression, which is not detectable at later stages of development. However in articular cartilage, collagen type-IIA protein does not appear in the interzones, which provides evidence for the belief that the cartilage anlagen that form individual skeletal elements are not continuous (Ng *et al*. 1993, Pitsillides & Ashhurst 2008). This suggests that the populations of cells which will become the growth and articular cartilage chondrocytes are discrete. Evidence for these discrete origins is also provided by the GDF5-mediated tracking of interzonal cells, and by the unique expression of versican, an extracellular matrix proteoglycan, in the regions of the presumptive articular cartilage in joint morphogenesis (Pacifici *et al*. 2005, 2006, Snow *et al*. 2005, Hyde *et al*. 2007).

Interzones first appear as densely cellular, homogenous regions with GDF5, WNT9A, autotaxin and chordin being known as interzone markers (Pacifici *et al*. 2005). Non-canonical WNT9A signalling is particularly important early in development as it acts to inhibit chondrocyte differentiation at the presumptive joint site (Hartmann & Tabin 2001). As joint development progresses, the interzone differentiates into three recognisable layers: two chondrogenic layers which cover the articular surfaces of the developing opposed skeletal elements and an intermediate layer which separates them. There is evidence to suggest that the cells derived from this intermediate layer differentiate to become articular chondrocytes, while the outer layer chondrocytes are incorporated into the growing epiphysis (Ito & Kida 2000).

Joint cavity formation is thought to occur due to a combination of extrinsic mechanical factors and intrinsic factors. Cell death is not thought to be primarily responsible for the formation of the joint cleft; rather, changes in extracellular matrix composition including hyaluronan synthesis, mediated by mechanical activation of the MEK–ERK pathway, which result in a loss of tissue cohesion have been implicated (Archer *et al*. 1994, Ito & Kida 2000, Bastow *et al*. 2005).

### Articular cartilage phenotype

Fully developed uncalcified articular cartilage can be divided into superficial, intermediate and deep zones (Fig. 1). These different zones consist of just one cell type, the chondrocyte. These chondrocytes differ from the transient growth plate chondrocytes as they maintain a stable phenotype characterised by small cell size and expression of tenascin-C, and they do not undergo a sequence of proliferation, maturation, hypertrophy, apoptosis and ossification (Pacifici *et al*. 2005, 2006). It has been suggested that the expression of the transcription factor ETS-related gene (ERG) is necessary early in joint development to establish the articular chondrocyte phenotype (Pacifici *et al*. 2006). It is the organisation of cells and their collagen type-II and aggrecan-rich matrix in the differing zones, however, which makes them distinct from one another. Whilst the superficial zone consists of elongated chondrocytes orientated parallel to the surface of the cartilage, chondrocytes in the deep zone are more rounded and aligned along the collagen fibrils. The collagen fibrils in the intermediate zone of the articular cartilage are arranged in arches which allow the transition from the deep and superficial zones (Minns & Steven 1977).

The deep zone of the articular cartilage forms an interface, termed the tidemark, with calcified cartilage which forms through mechanisms that are not quite understood. Within this calcified cartilage, the chondrocytes are hypertrophic and this is accompanied by their expression of collagen type-X and alkaline phosphatase (Heinegard & Oldberg 1989, Gannon *et al*. 1991, Stephens *et al*. 1992). Therefore, despite the cellular singularity and stability of the articular cartilage chondrocyte, there is some degree of transiency due to the varying chondrocyte phenotypes which exist within the entirety of the tissue. This notion is supported by the presence of chondrocyte progenitors in the superficial zone both at young stages of development and in the mature cartilage (Dowthwaite *et al*. 2004). Therefore tighter management of these dynamic events, although much slower than those in the growth plate, may alone underpin the maintenance of the articular cartilage stability.

The calcified cartilage layer is semipermeable and whilst it acts as a physical barrier for vascular invasion of the overlying articular cartilage, it does permit the passage of small molecules from the underlying subchondral bone (Arkill & Winlove 2008). In humans, the calcified cartilage thickness varies widely, from ∼20 to 250 μm however, like the tidemark, this a dynamic structure which can be remodelled during ageing and disease, as highlighted by observed morphological changes in osteoarthritis including tidemark duplication and increased calcified cartilage thickness (Lane & Bullough 1980, Oettmeier *et al*. 1989, Oegema *et al*. 1997, Hunziker *et al*. 2002, Burr 2004). The molecular mechanisms regulating events at this osteochondral interface are yet to be fully defined. Their identification may provide potential targets for therapeutic intervention.

## The ectopic initiation of cartilage–bone transitions

The transition of cartilage to bone in the healthy individual is under tight regulation so as to prevent disturbed development and/or longitudinal bone growth. Observations from disorders such as hypothyroidism, rickets and dwarfism may provide insights into the mechanisms underpinning defective hypertrophic differentiation and ossification (Combs *et al*. 2011, Santos *et al*. 2013). Regulation is also observed in repair of fractured bone tissue, in which there is a deliberate re-initiation of the endochondral processes (see above) previously discussed in this review. There has been much recent focus upon the WNT signalling pathway in fracture repair and, in particular, the recent discovery of enhanced repair through the administration of neutralizing antibodies against sclerostin, a known WNT inhibitor (Secreto *et al*. 2009, Ominsky *et al*. 2011, Virk *et al*. 2013). In contrast to this desired acceleration in endochondral ossification processes, in certain diseases the ectopic redeployment of these processes is detrimental and considered to be at least contributory to the observed disease pathology. Herein we discuss conditions in which this occurs and touch upon current understanding regarding the role of these processes in their aetiology.

### Osteoarthritis

**Figure 2:**
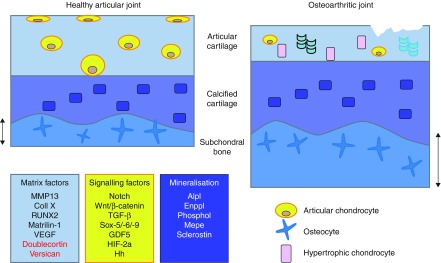
Schematic diagram depicting the healthy articular joint and the osteoarthritic joint in which articular cartilage fibrillation and degradation is observed with concomitant subchondral bone thickening. Contributing to this osteoarthritic pathology, the normally ‘stable’ articular chondrocytes of the articular cartilage adopt a ‘transient’ phenotype with observed chondrocyte hypertrophy and matrix mineralisation similar to that seen in the growth cartilage depicted in Fig. 1. Potential regulation of these processes may include changes in the expression of matrix factors (blue box; factors induced in osteoarthritis development in black, factors lost in red), signalling pathways affecting chondrocyte phenotype and function (yellow box) and the known regulators of mineralisation processes (purple box) (Grover & Roughley 1993, Pacifici *et al*. 2006, Zhang *et al*. 2007, Fosang & Beier 2011, Pitsillides & Beier 2011, Staines *et al*. 2012*b*). Lessons may be learnt from these and their application may better identify disease aetiology.

Osteoarthritis is a degenerative joint disease and a massive world-wide healthcare burden. Characterised by articular cartilage loss, subchondral bone thickening and osteophyte formation, the osteoarthritic joint afflicts much pain and disability on its sufferers. Its underpinning molecular mechanisms are, nevertheless, not fully understood; indeed it is even still a matter of debate as to which is the preceding pathology. However, there is increasing evidence implicating the re-initiation of the transient chondrocyte phenotype in osteoarthritic aetiology and pathology (Fosang & Beier 2011, Pitsillides & Beier 2011; Fig. 2). This notion is based upon the previously discussed common embryonic development of cartilage and bone and the current evidence certainly merits thorough examination. However, it has been met with some controversy in the field (Brew *et al*. 2010). Such controversies may potentially be addressed through a better understanding of the articular cartilage volume to surface area ratios in different species, and thus may provide some explanation as to why the degree of chondrocyte transiency in the articular cartilage differs from species to species.

In osteoarthritic cartilage, there have been observed decreases in collagen type-II and aggrecan integrity in comparison to normal articular cartilage (Helminen *et al*. 1993, Garnero *et al*. 2002, Jalba *et al*. 2011, Henrotin *et al*. 2013). Furthermore, markers previously thought to be unique to the hypertrophic chondrocytes of the growth plate have been detected in the uncalcified articular cartilage in both animal models of osteoarthritis, and in patients with the disease. The best recognised of these are MMP13 and collagen type-X, however, also detected are alkaline phosphatase, osteopontin, IHH and osteocalcin (Hoyland *et al*. 1991, Aigner *et al*. 1993, Pullig *et al*. 2000, Appleton *et al*. 2007, Studer *et al*. 2012). As in the transient growth plate cartilage, chondrocyte hypertrophy is considered to be a prerequisite for matrix mineralisation and in osteoarthritis, the increased formation of hydroxyapatite has been documented and is consistent with hypertrophic chondrocyte changes (Fuerst *et al*. 2009).

MMP13, a key marker of chondrocyte hypertrophy, is proving a critical target in osteoarthritis research due to its potent role in the degradation of collagen type-II, proteoglycans, collagen types-IV and IX, osteonectin and perlecan (Wang *et al*. 2013). Indeed, Mmp13-deficient mice predictably have an endochondral bone growth defect with an expanded hypertrophic chondrocyte zone which was not attributable to increased chondrocyte proliferation, increased proteoglycan turnover and increased trabecular bone (Stickens *et al*. 2004). Nevertheless, whilst the surgical induction of osteoarthritis in this mouse causes chondrocyte hypertrophy and osteophyte formation, the graded score of cartilage degradation was intriguingly significantly reduced (Little *et al*. 1999). Deletion of the *Mmp13* gene specifically in chondrocytes also produces similar deceleration of osteoarthritis disease progression following meniscal-ligamentous injury in a mouse model (Wang *et al*. 2013). Consistent with this crucial role for *Mmp13*, its overexpression was also found to result in pathological osteoarthritis-like changes in the articular cartilage of mice (Neuhold *et al*. 2001). Recent research has implicated the WNT signalling pathway and its inhibitors as important regulators of *MMP13* (van den Bosch *et al*. 2013, Chan *et al*. 2013, Staines *et al*. 2013), which is consistent with critical WNT function in the prevention of joint pathologies (Zhu *et al*. 2008, 2009, Miclea *et al*. 2011).

Epigenetics has been strongly implicated in osteoarthritis in recent years with all three currently known mechanisms – DNA methylation, histone modifications and non-coding RNAs – demonstrate a capacity to control the chondrocyte phenotype. This too has highlighted a role for *MMP13*. More specifically, *MMP13* promotor methylation is altered in osteoarthritic cartilage, suggesting that these epigenetic changes can therefore drive the chondrocyte hypertrophy observed in the pathological state (Roach *et al*. 2005). It should be noted that the precise initiation and control of these events is yet to be established. However, with the surge in epigenetic studies in recent years, it is certainly an exciting and promising time for this field.

This evidence therefore pinpoints the need to decipher the molecular mechanisms underpinning the recapitulation of some of these developmental ‘hallmarks’ by articular cartilage chondrocytes in osteoarthritis. It is envisaged that this will undoubtedly advance understanding into disease pathology and might ultimately define whether the pathogenic pathways in osteoarthritis can be prevented by regulating the stability of chondrocytes by limiting acquisition of their transient, growth-related phenotype.

Certainly the field of tissue engineering would benefit from such understanding. Combining the use of cells and biomaterials, this approach has emerged as a promising target for cartilage repair. The first repair of cartilage defects was described in 1994 and used autologous chondrocytes (Brittberg *et al*. 1994). Despite the limitations associated with this, including the need to disrupt healthy cartilage and problems with culturing chondrocytes, its emergence has certainly set the platform from which cartilage tissue engineering has greatly progressed in recent years. The use of human mesenchymal stem cells, with their ability to overcome the limitations defined by the use of chondrocytes, has been repeatedly reported in osteoarthritis as a means of providing promise and much excitement in the field (Luyten 2004, Coleman *et al*. 2010). It has become apparent that human mesenchymal stem cells can also gain expression of the chondrocyte hypertrophy marker, collagen type-X, and it is clear that this still limits their effectiveness as a candidate for tissue engineering. Indeed, a recent study has shown that mesenchymal stem cells express higher levels of the genes associated with osteoarthritis, upon culture, than do chondrocytes derived from the osteoarthritic joints themselves (Mwale *et al*. 2010, Fernandes *et al*. 2013).

### Intervertebral disc calcification

Intervertebral disc (IVD) degeneration is a major cause of back pain worldwide with complex, expensive surgery which is often prone to failure (Urban & Roberts 1995). Located between the vertebral bodies of the spine, the cartilaginous IVD functions to resist compressive loads (Broberg 1983). Anatomically, it consists of a central nucleus pulposus contained within a fibrocartilage ring, the annulus fibrosus, laterally and the cartilage end plates inferiorly and superiorly (Roberts *et al*. 2006). The nucleus pulposus shares many similarities with articular cartilage, both in its matrix composition and its cellular metabolism (Raj 2008). Associated with ageing and with abnormal mechanical loading playing a substantial role, the pathogenesis of IVD degeneration is yet to be fully elucidated. There is, however, emerging evidence for the occurrence of hypertrophic differentiation in this process.

The IVDs of patients with degenerative disc disease have increased alkaline phosphatase activity, and also in contrast to healthy IVDs express significant levels of hypertrophic differentiation markers, including collagen type-X, osteoprotegerin (OPG), MMP13 and RUNX2 (Boos *et al*. 1997, Nerlich *et al*. 1997, Sato *et al*. 2008, Rutges *et al*. 2010, Hristova *et al*. 2011). Associated with this, microCT analysis clearly shows increased levels of IVD calcification with increasing degeneration (Rutges *et al*. 2010). A recent *in vitro* study has sought to examine the mineralisation potential of the cells of the annulus fibrosus, and concluded that under certain conditions these cells can induce mineralisation, as indicated by their increased von Kossa staining, alkaline phosphatase, MMP13 and RUNX2 expression (Nosikova *et al*. 2013).

Understanding of the mechanisms supporting induction of such hypertrophy in IVD cells is somewhat lacking. However, it was shown recently that PTH can enhance disc repair through its inhibition of collagen type-X and alkaline phosphatase expression, and through the promotion of collagen type-II production. These regenerative properties of PTH are mediated through MAPK signalling (Madiraju *et al*. 2013). The authors of this study suggest that PTH supplementation may prevent further calcification in degenerated discs and potentially enhance other cell-based therapies. This is certainly promising due to the known critical role for PTH signalling in chondrocyte hypertrophy and matrix mineralisation (Mackie *et al*. 2011, Yano *et al*. 2013). The current major problem hindering IVD tissue engineering, like in osteoarthritis, is the undesirable expression of collagen type-X by human mesenchymal stem cells. Recent studies have highlighted that PTH treatment can also inhibit such misexpression and may thus open additional avenues for the treatment of degenerative IVDs with mesenchymal stem cells (Mwale *et al*. 2010).

### Heterotopic ossification

Heterotopic ossification (HO) is a common occurrence in muscle, tendon and ligaments following trauma by injury, disease or surgery. Initiated by cartilage formation, the endochondral ossification and ectopic bone formation in these tissues can produce severe functional impairment (Medici & Olsen 2012).

Besides the trauma-induced HO described, a rare disease called fibrodysplasia ossificans progressiva (FOP) is a hereditary form of HO, presenting itself as painful and highly inflammatory soft tissue swellings which progressively ossify rendering the sufferer immobile (Cohen *et al*. 1993, Shore *et al*. 2006, Medici & Olsen 2012). Like in trauma-induced HO, the aberrant bone formation in patients with FOP occurs by means of endochondral ossification processes. Emerging evidence has strongly implicated increased bone morphogenetic protein (BMP) signalling in the pathogenesis of FOP, as well as in trauma-induced HO (Cohen *et al*. 1993, Kwapisz *et al*. 2013). The identification of ACVR1/ALK2, one of the four type-I receptors that mediate BMP signalling, as the mutated gene in FOP in 2006 has further highlighted the BMP signalling pathway as an attractive target for future therapy (Shore *et al*. 2006).

## Conclusions

The aberrant redeployment of embryonic processes in diseases such as osteoarthritis and IVD degeneration is now well established. Emerging evidence has further fuelled the hypothesis that lessons for limiting disease progression can be acquired from the regulators of transient cartilage biogenesis and development. This review provides some pointers as to potential targets for future drug therapies or tissue engineering approaches which will further our understanding of the underpinning molecular mechanisms involved in these diseases, and will provide advances towards patient benefit.
